# Metformin Ameliorates Inflammatory Bowel Disease by Suppression of the STAT3 Signaling Pathway and Regulation of the between Th17/Treg Balance

**DOI:** 10.1371/journal.pone.0135858

**Published:** 2015-09-11

**Authors:** Seon-Yeong Lee, Seung Hoon Lee, Eun-Ji Yang, Eun-Kyung Kim, Jae-Kyung Kim, Dong-Yun Shin, Mi-La Cho

**Affiliations:** 1 The Rheumatism Research Center, Catholic Research Institute of Medical Science, The Catholic University of Korea, Seoul, South Korea; 2 The Laboratory of Immune Network, CRCID, The Catholic University, Seoul, Republic of Korea; 3 College of Pharmacy, Gachon University of Medicine and Science, 191 Hambakmoe-ro, Yeonsu-gu, Incheon 406–799, Korea; Jackson Laboratory, UNITED STATES

## Abstract

**Objective:**

Metformin is used to treat type 2 diabetes. We sought to determine whether metformin reduces inflammation, by regulating p-signal transducer and activator of transcription 3 (STAT3) expression and T-helper 17 (Th17) cell proliferation, in a mouse model of inflammatory bowel disease (IBD).

**Methods:**

IBD mice were administered metformin for 16 days and their tissues were analyzed. AMP-activated protein kinase (AMPK), the mammalian target of rapamycin (mTOR), p-STAT3 and p-STAT5 in the spleen and lymph nodes were detected using immunohistochemistry and confocal microscopy. Gene expression was determined using quantitative PCR assays, and protein expression levels were measured using western blotting and enzyme-linked immunosorbent assays. Human HT-29 cell proliferation was evaluated using MTT assays.

**Results:**

Metformin reduced disease activity index scores and inhibited weight loss. Metformin also decreased the colonic histological score and inflammatory mediators and increased colon lengths increased. Treatment with metformin inhibited the expression of interleukin (IL)-17, p-STAT3, and p-mTOR. In contrast, metformin treatment increased expression levels of p-AMPK and Foxp3. In addition, expression of inflammatory cytokines decreased in a dose-dependent manner in inflamed human HT-29 cells cultured with metformin at various concentrations.

**Conclusions:**

Metformin attenuates IBD severity and reduces inflammation through the inhibition of p-STAT3 and IL-17 expression. Our results have increased our understanding of this chronic inflammatory disease, and support the strategy of using p-STAT3 inhibitors to treat IBD.

## Introduction

The gastrointestinal tract has a central role in the regulation of immune responses against pathogens. Inflammatory bowel disease (IBD), an autoimmune disease characterized by immune inflammatory responses in the gastrointestinal tract, causes instability of the human gut and an uncontrolled inflammatory response. This chronic and relapsing disease induces unintended weight loss, diarrhea, and rectal bleeding [1,2].

The pathogenesis of IBD is complex, but the relevance of T helper (Th) 17 cells and interleukin (IL)-17 to IBD pathogenesis has been suggested in previous preclinical and clinical investigations [3,4]. Upregulation of Th17 cell proliferation and IL-17 expression is associated with several autoimmune diseases, including IBD.

When the proinflammatory cytokine IL-17 is expressed by Th17 cells, an inflammatory response is triggered, thereby inducing the activation of phosphorylated signal transducer and activator of transcription 3 (p-STAT3) [5,6]. Since STAT3 is a transcription factor that regulates a large number of proinflammatory cytokines [7], inhibition of STAT3 activation has been demonstrated as a promising target for several autoimmune diseases. Inhibitors of p-STAT3 ameliorate experimental autoimmune diseases by promoting regulatory T (Treg) cell proliferation [8,9]. Accumulating evidence indicates that inhibition of p-STAT3 has an anti-inflammatory effect and reduces Th17 cell proliferation [10,11]. Thus, the balance between Th17 and Treg cells plays an important role during an inflammatory response. It has been suggested that the balance between Th17 and Treg cells is adversely affected in several autoimmune disorders, including IBD, and that this imbalance enhances chronic and immoderate inflammation [12–14].

Metformin was originally used to treat type 2 diabetes. The pharmacological activity of metformin is dependent on its ability to induce AMP-activated protein kinase (AMPK) [4]. Metformin exerts anti-inflammatory functions by inhibiting the activation of NF-κB and enhancing the activation of AMPK [15–17]. AMPK is an upstream kinase of mammalian target of rapamycin (mTOR), and also an inhibitor of the mTOR pathway [18,19]. Recently, metformin was shown to inhibit inflammation, and reduce the expression of IL-17 and p-STAT3 in experimental autoimmune disease mice [20].

We hypothesized that metformin inhibits the expression of proinflammatory cytokines and chemokines during the colonic inflammatory response. The aim of our study was to investigate the anti-inflammatory activity of metformin in IBD mice by investigating its effects on the inhibition of p-STAT3 and IL-17 expression.

## Materials and Methods

### Animals

We purchased C57BL/6 mice (8-weeks-old) from SLC Inc. (Shozuoka, Japan) and maintained them under specific pathogen-free conditions at the Institute of Medical Science (Catholic University of Korea). Mice were provided standard mouse chow (Ralston Purina, St. Louis, MO, USA) and water ad libitum. All experimental procedures were approved by the Animal Research Ethics Committee of the Catholic University of Korea, which conformed to all National Institutes of Health of the USA guidelines. All surgeries were performed under isoflurane anesthesia and we made an effort to minimize the suffering of all animals. Mice were euthanized at the end of a study for the purpose of sample collection and histologic examination by CO_2_ chamber. The experimental protocol was approved, and all animals were treated and sacrificed in accordance with the guidelines of the Catholic university of Korea on Use and Care of Animals.

### Induction of IBD and metformin treatment

Colitis was induced in C57BL/6 mice through the oral ingestion of 2.5% dextran sulfate sodium (DSS; MP Biomedicals, Santa Ana, CA, USA) for 4 days. Mice were intrarectally injected with metformin (50 mg/kg) daily for 16 days after IBD induction. During DSS and metformin treatment, the body weight of mice were monitored daily.

### Assessment of inflammation

During the experimental period, severity of colitis was assessed daily by measuring the percentage of body weight change and disease activity index (DAI). The DAI was calculated as previously described, with the score incorporating indicators of body weight loss (0 points, <5% weight loss; 1 point, 5–10% weight loss; 2 points, 10–15% weight loss; 3 points, 15–20% weight loss; and 4 points, >20% weight loss), stool consistency (0 points, pellets; 2 points, paste-like/semi-formed stool; and 4 points, liquid stool), and rectal bleeding (0 points, no rectal bleeding; 2 points, hemoccult-positive; and 4 points, visible gross bleeding).

### Histopathological analysis and immunohistochemistry

Formalin-fixed paraffin-embedded colon tissue sections (7-μm thickness) were stained with hematoxylin and eosin (H&E). Assessment included reporting of edema, extent of injury, leukocyte infiltration, crypt abscesses, and loss of goblet cells. In this grading system, inflammation severity was scored using a scale of 0–3 (0, no inflammation; 1, slight inflammation; 2, moderate inflammation; and 3, severe inflammation), as the extent of injury (0, no injury; 1, mucosal injury; 2, mucosal and submucosal injury; and 3, transmural injury). Crypt damage was scored using a scale of 0–4 (0, no damage; 1, basal third was damaged; 2, basal two-thirds was damaged; 3, only the surface epithelium was intact; and 4, loss of entire crypt and epithelium). Each value was multiplied by an extent index, ranging from 1–4, that reflected the amount of involvement for each section (1, 0–25%; 2, 26–50%; 3, 51–75%; and 4, 76–100%). At least three sections from each colon were analyzed.

Sections were treated with 3% (v/v) H_2_O_2_ in methanol to block endogenous peroxidase activity. Immunohistochemistry (IHC) was performed using the Vectastain ABC kit (Vector Laboratories, Burlingame, CA, USA). Tissue sections were incubated with antibodies against tumor necrosis factor alpha (TNF-α), vascular endothelial growth factor (VEGF), IL-1β, IL-8 (all from Santa Cruz Biotechnology, Santa Cruz, CA, USA), and IL-6 (Abcam) overnight at 4°C. Sections were then incubated with a biotinylated secondary antibody and a streptavidin–peroxidase complex for 1 h. Color complexes were developed using 3,3'-diaminobenzidine (DAKO, Carpinteria, CA, USA). Histological assessments were conducted by two independent blinded observers. The independent observers involved in analysis of immunohistochemistry staining count 3 microscopy photos.

Images were captured using a DP71 digital camera (Olympus, Center Valley, PA, USA) attached to an Olympus BX41 microscope (Olympus, Japan) at a magnification of 3400×.

### Western blotting

Colonic cells isolated from control or metformin-treated mice were washed with cold saline, and total proteins extracted with lysis buffer [1% Nonidet P-40, phenylmethanesulfonylfluoride (PMSF), 2 mM sodium vanadate, 0.1% sodium deoxycholate, and a protease inhibitor mixture; Roche Applied Science, Mannheim, Germany]. Harvested lysates were centrifuged (15 min, 4°C) to pellet cellular debris. Proteins were loaded onto 10% polyacrylamide gels and subjected to sodium dodecyl sulfate polyacrylamide gel electrophoresis, and then transferred to nitrocellulose membranes (Invitrogen Life Technologies, Carlsbad, CA, USA). Membranes were blocked with 5% (w/v) non-fat milk in Tris-buffered saline with 0.1% Tween-20 (TBST) for 1 h at room temperature and incubated with antibodies against p-STAT3 Y705, p-STAT3 S727, p-AMPK, p-mTOR, p-AMPK, and β-actin overnight at 4°C. Membranes were then incubated with goat anti-mouse or goat anti-rabbit horseradish peroxidase-conjugated antibodies. Immunoreactivity was determined using enhanced chemiluminescence reagents (Amersham Biosciences, Piscataway, NJ, USA).

### Quantitative polymerase chain reaction (qPCR) analysis of gene expression

Total RNA was extracted using TRIzol reagent (Molecular Research Center, Cincinnati, OH, USA). The concentration of RNA in each sample was measured using a NanoDrop ND-1000 (Thermo Fisher Scientific, MA, USA). Total RNA (2 μg) was reverse transcribed into cDNA using the Transcriptor First Strand cDNA Synthesis Kit (Roche Applied Science). Expression of mRNAs was estimated using qPCR assays with FastStart SYBR Green Master (Roche Applied Science) using StepOnePlus (Applied Biosystems) according to the manufacturer’s instructions.

### Confocal microscope

Spleen or mesenteric lymph node tissue sections (7-μm thickness) were incubated with phycoerythrin-conjugated antibodies against IL-17, p-STAT3 Y705, p-STAT3 S727, Foxp3, an allophycocyanin-conjugated antibody against CD25, a fluorescein isothiocyanate-conjugated antibody against CD4 (all from eBioscience, San Diego, CA, USA), or unconjugated rabbit antibodies against mTOR, AMPK, or P53 (all from Cell Signaling Technology) overnight at 4°C. Tissue sections were then washed with phosphate-buffered saline and incubated with the appropriate secondary antibodies conjugated with fluorescent molecules where necessary. Sections were analyzed using a Zeiss microscope (LSM 510 Meta; Carl Zeiss, Oberkochen, Germany) at a magnification of 3400×.

### Cell treatments

HT29 human colon cells (ATCC, VA, USA) were cultured in RPMI 1640 medium at 37°C/5% CO_2_. We treated 2 × 10^5^ HT29 cells with 0.2 or 1 mM metformin (Sigma-Aldrich, St Louis, MO, USA).

### Determination of VEGF concentration

The concentrations of VEGF in HT29 culture supernatants were measured using a specific sandwich enzyme-linked immunosorbent assay (ELISA; R&D Systems, Minneapolis, MN, USA). Absorbance was measured at 405 nm on an ELISA microplate reader (Molecular Devices, Sunnyvale, CA, USA). The cytotoxicity of chemical inhibitors against HT29 cells was evaluated using 3-(4,5-dimethylthiazol-2-yl)-2,5-diphenyltetrazolium bromide (MTT) assays.

### Statistical analysis

All data are expressed as means ± standard deviation (SD). Statistical analysis was performed with SPSS version 10.0 for Windows (SPSS Inc., Chicago, IL, USA). Differences between groups were analyzed using an unpaired Student’s *t*-test, assuming equal variances. A *P-*value less than 0.05 was considered statistically significant.

## Results

### Metformin suppressed induction of IBD

**Fig 1:**
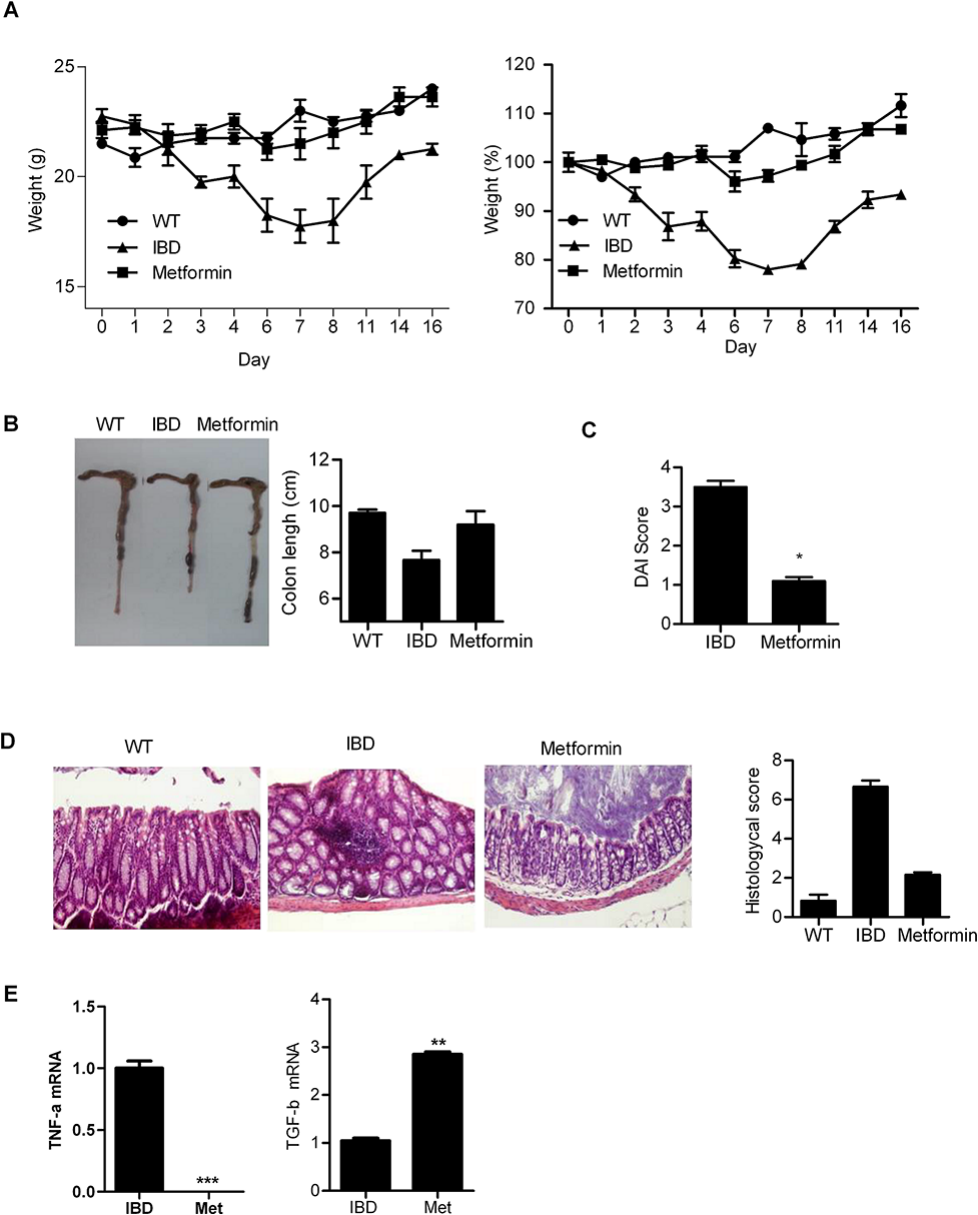
Metformin prevents induction of inflammatory bowel disease (IBD). Metformin (1 mg/mouse) was administered daily to mice with IBD for 16 days. (A) Weight of mice with IBD. (B and C) disease activity index (DAI) score and colon length. (D) Histopathlogical analysis showed tissue degradation in mice with IBD, and metformin-treated IBD mice. All tissue sections were counterstained with hematoxylin (100× magnification). The scale bar indicates 50 μm. (E) mRNA levels of tumor necrosis factor alpha (TNF-α) and transforming growth factor beta (TGF-β) in the colon tissues of IBD mice and metformin-treated IBD mice were determined by qPCR. Data are presented as the mean ± SD from three independent experiments (*P < 0.05, **P < 0.03, ***P < 0.001).

Treatment with metformin prevented IBD in mice, with their weight (Fig 1A) and colon length (Fig 1C) unaffected. The DAI scores in mice with IBD significantly decreased (Fig 1B), and colon tissues were degraded (Fig 1D). Levels of TNF-α increased in IBD [21], and transforming growth factor beta (TGF-β) is associated with the occurrence of IBD [22]; therefore, we examined the mRNA expression levels of TNF-α and TGF-β. TNF-α gene expression significantly reduced in the colon tissue of metformin-treated mice compared with that in control mice. The mRNA expression levels of TGF-β markedly increased in the colon tissue of metformin-treated mice compared with that in control mice (Fig 1E).

### Metformin suppressed IBD by inhibiting inflammatory cytokines and mTOR/STAT3

**Fig 2:**
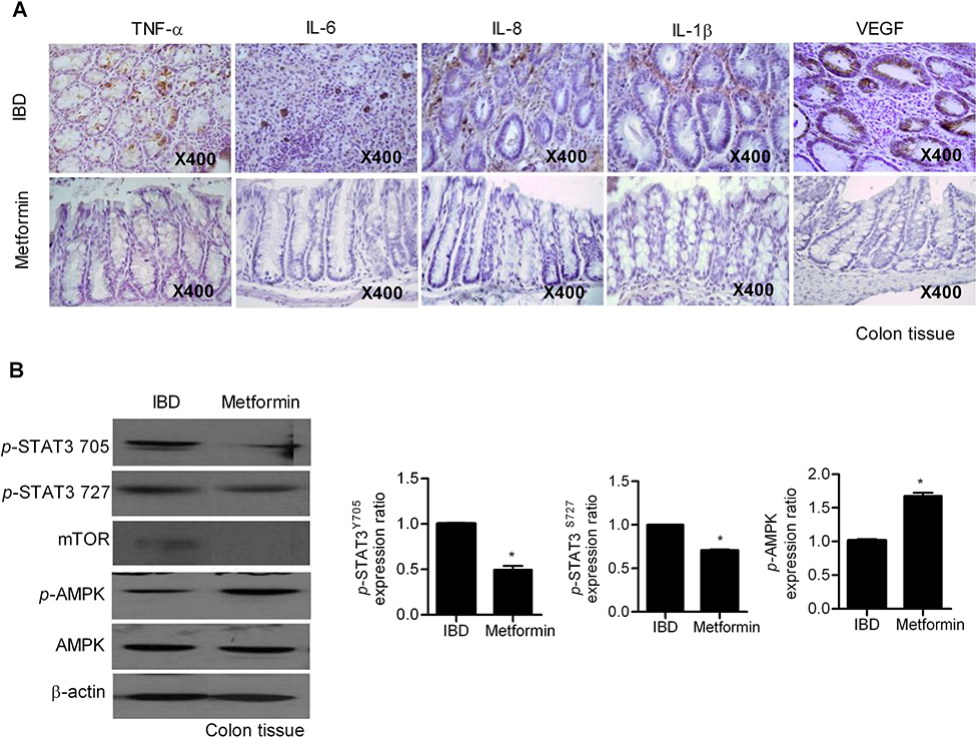
Metformin treatment suppresses inflammation and p-signal transducer and activator of transcription 3 (STAT3) expression, but enhances the expression of AMP-activated protein kinase (AMPK) in a mouse model of inflammatory bowel disease (IBD). (A) Immunohistochemical detection of interleukin (IL)-1β, IL-6, IL-8, TNF-α, and vascular endothelial growth factor (VEGF) in the colon tissues of IBD mice and metformin-treated IBD mice. All tissue sections were counterstained with hematoxylin (400× magnification). The scale bar indicates 50 μm. (B) Expression of p-STAT3, p-mTOR, and p-AMPK proteins were determined by western blotting. Data are presented as the mean ± SD from three independent experiments (*P < 0.05, **P < 0.03).

To investigate the effects of metformin on colon tissue inflammation, we conducted immunohistochemical staining to visualize proinflammatory cytokines in tissue sections. Expression of TNF-α, IL-1β, IL-8, IL-6, and VEGF decreased in metformin-treated mice (Fig 2A). Excessive mTOR-STAT3 signaling is associated with IBD pathogenesis and is persistently detected in patients with IBD [23]. Western blotting analysis revealed that treatment with metformin significantly activated AMPK, but significantly suppressed p-STAT3 expression (Fig 2B).

### Metformin regulates the differentiation of Th17 and Treg cells

**Fig 3:**
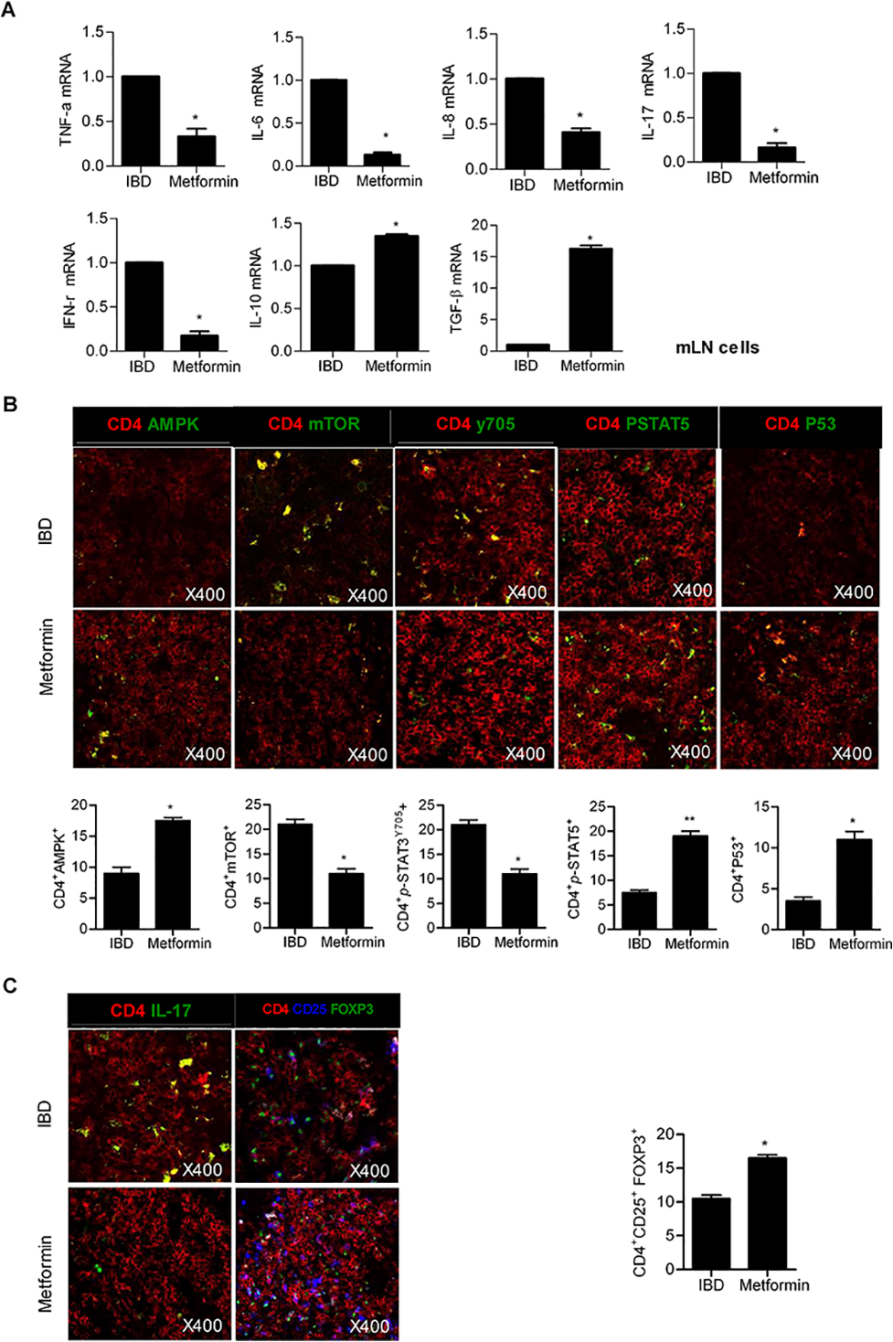
Metformin controls mRNA expression levels of inflammatory cytokines and the differentiation of Th17 and Treg cells through the regulation of signal transducer and activator of transcription 3 (STAT3) and STAT5 phosphorylation in lymph nodes. (A) mRNA levels of proinflammatory cytokines [tumor necrosis factor (TNF)-α, interleukin (IL)-6, IL-8, IL-17, and interferon (IFN)-γ] decreased in the lymph nodes of metformin-treated inflammatory bowel disease (IBD) mice. (B) Lymph node tissue sections from IBD and metformin-treated IBD mice were subjected to immunostaining to determine the presence of CD4^+^IL-17^+^ or CD4^+^CD25^+^Foxp3^+^ cells. Data are presented as the mean ± SD from three independent experiments (*P < 0.05, **P < 0.03).

**Fig 4:**
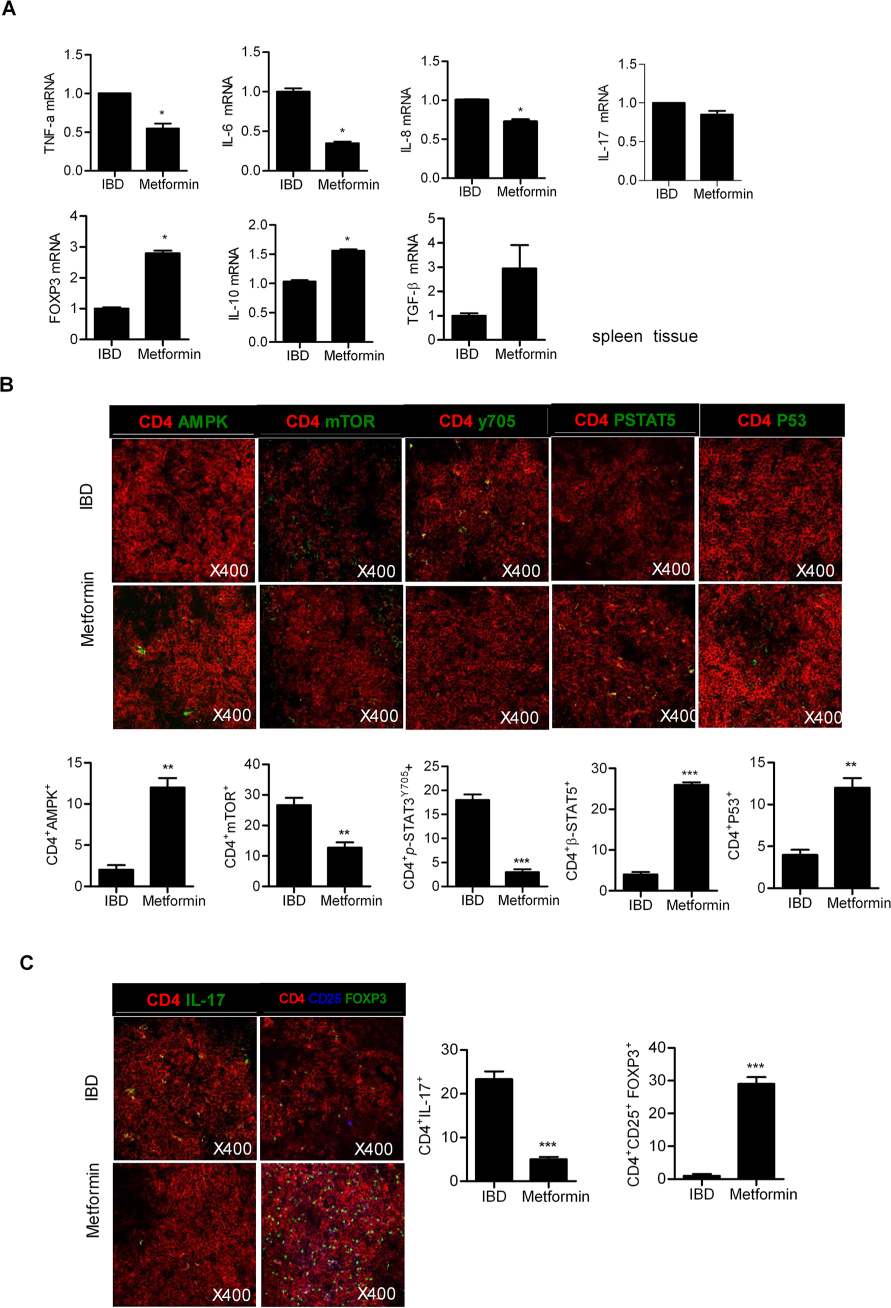
Metformin controls the mRNA expression levels of inflammatory cytokines and the differentiation of Th17 and Treg cells through the regulation of signal transducer and activator of transcription 3 (STAT3) and STAT5 phosphorylation in the spleen. (A) mRNA levels of proinflammatory cytokines [tumor necrosis factor (TNF)-α, interleukin (IL)-6, IL-8, IL-17, and interferon (IFN)-γ] decreased in the spleens of metformin-treated inflammatory bowel disease (IBD) mice. (B) Spleen tissue sections of IBD and metformin-treated IBD mice were subjected to immunostaining to determine the presence of CD4^+^IL-17^+^ or CD4^+^CD25^+^Foxp3^+^ cells. Data are presented as the mean ± SD from three independent experiments (*P < 0.05, **P < 0.03).

Th17 cells expressing IL-17 are regulated by STAT3 activation, while Treg cells producing forkhead box P3 (FOXP3) are controlled by STAT5 activation. Th17 and Treg cells play a significant role in IBD; Th17 cells infiltrate the gastrointestinal tract of patients with IBD to a massive extent [4], while Treg cell deficiency results in IBD [24]. In addition, when the proliferation of Treg cells is downregulated, Th17 cell proliferation is promoted in the peripheral blood of patients with IBD [25]. The balance between Th17 and Treg cell proliferation levels is an important factor in designing therapies for IBD. The mRNA expression levels of proinflammatory and anti-inflammatory cytokines were measured by qPCR assays. The mRNA levels of proinflammatory cytokines, such as TNF-α, IL-6, IL-8, IL-17 and interferon (IFN)-γ significantly decreased after metformin treatment. However, the mRNA expression levels of anti-inflammatory cytokines, such as IL-10 and TGF-β, were significantly enhanced by metformin treatment (Figs 3A and 4A). Expression levels of mTOR, p-STAT3 and IL-17 diminished in the lymph nodes and spleens of IBD mice treated with metformin. In contrast, expression of AMPK, p-STAT5, P-53 and FOXP3 increased in IBD mice treated with metformin (Figs 3B and 4B). The number of CD4^+^ cells expressing AMPK, mTOR, p-STAT3 and IL-17 significantly reduced in metformin-treated mice compared with control IBD mice (Fig 3B and 3C).

### Metformin affects inflammation in human colon cells

**Fig 5:**
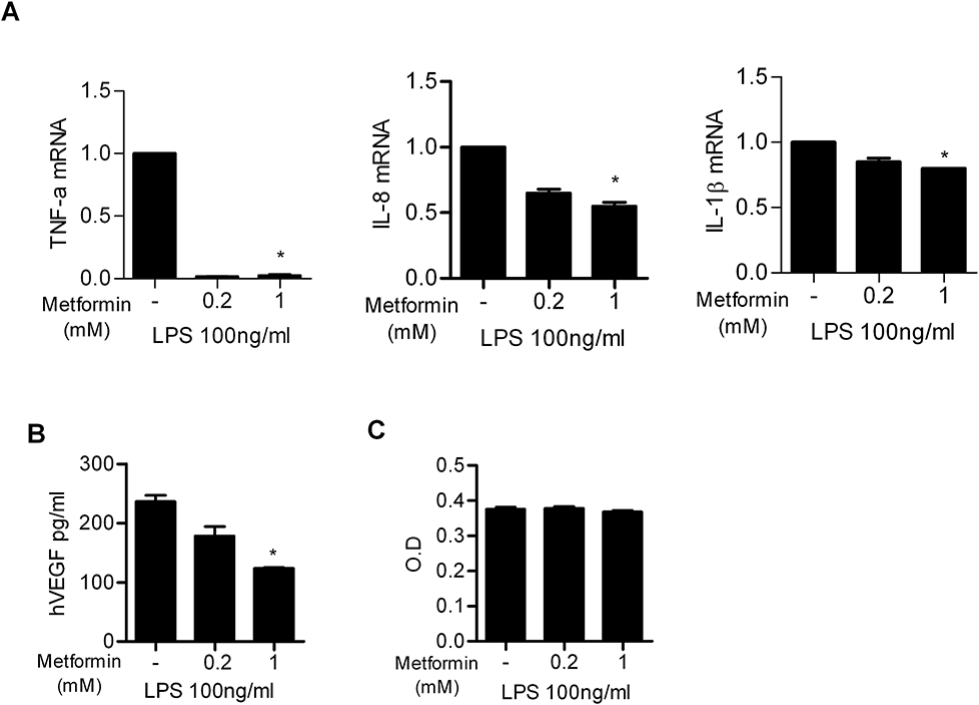
Metformin treatment reduces the mRNA levels of proinflammatory cytokines in human HT-29 cells. (A) Tumor necrosis factor (TNF)-α, interleukin (IL)-1β, and IL-8 mRNA expression levels decreased in human HT-29 cells stimulated with LPS and then treated with metformin. (B) The production of vascular endothelial growth factor (VEGF) decreased in human HT-29 cells stimulated with lipopolysaccharide (LPS) and then treated with metformin. (C) Cell viability was not altered by various concentrations of metformin. Data are presented as the mean ± SD from three independent experiments (*P < 0.05, **P < 0.03, ***P < 0.001).

To assess whether metformin can inhibit the inflammatory response in human colon cells, we used qPCR assays to determine proinflammatory cytokine expression levels. Inflammation was induced in cells of the human HT-29 cell line using lipopolysaccharide (LPS) and the cells were treated with metformin. The mRNA expression levels of inflammatory cytokines, including TNF-α, IL-1β, and IL-8 significantly decreased. VEGF expression in culture supernatants reduced significantly in a dose-dependent manner following treatment of cells with metformin (Fig 5A and 5B). We used MTT assays to identify whether the effects induced by metformin were owing to its toxicity. We observed no significant changes in cell viability following treatment with LPS or metformin (Fig 5C).

## Discussion

Metformin is widely used as an anti-diabetic drug, however there is little mechanistic evidence of its function with respect to the immune inflammatory response in IBD. To the best of our knowledge, the therapeutic functions of metformin *via* suppression of p-STAT3 in IBD have not been reported. In the current study, we revealed the anti-inflammatory functions of metformin in an IBD mouse model, which could aid in the development of therapeutic strategies against IBD.

The anti-inflammatory activity of metformin induced the inhibition of p-STAT3 expression. It has been demonstrated that p-STAT3 expression leads to increased levels of proinflammatory cytokines such as IL-6, IL-1β, and TNF-α [26]. Expression of IL-1β, IL-6, IL-8, VEGF, and TNF-α is also upregulated in IBD [27–29]. A significant reduction in proinflammatory cytokines (TNF-α, VEGF, IL-6, IL-8, and IL-1β) was observed in the colon tissue of metformin-treated IBD mice. Inhibition of proinflammatory cytokine and p-STAT3 expression is an important requirement for suppressing the inflammatory response. Our results show that metformin exerts anti-inflammatory effects in IBD.

AMPK and mTOR play an important role in protein synthesis and transcription. AMPK reduces mTOR activation, which in turn induces p-STAT3 expression [19,30]. AMPK activation induced by metformin inhibits mTOR activation and promotes p53 expression [31]. In this study, expression of AMPK and p53 increased in metformin-treated IBD mice. However, mTOR and p-STAT3 expression was downregulated by metformin treatment, therefore we concluded that metformin regulates the mTOR/STAT3 axis in an IBD mouse model.

Inflammation is a coordinated response to harmful stimuli, with the purpose of returning a system to its normal state. Chronic and excessive inflammation results in autoimmune disorders that are induced by increases in IL-17 expression levels. Th17 cells that produce IL-17 induce inflammation, but Treg cells expressing FOXP3 exert anti-inflammatory activity [32,33]. It is known that p-STAT3 inhibits Treg cell proliferation [34], while the activation of STAT5 sustains FOXP3 expression in Treg cells [35]. Our results show that metformin suppressed p-STAT3 expression, and promoted the expression of p-STAT5 and FOXP3. Our results suggest that metformin is involved in the anti-inflammatory response by regulating expression of p-STAT3/p-mTOR and p-STAT5.

We suppressed the expression of proinflammatory cytokines *in vitro* using metformin. The mRNA levels of IL-1β, IL-8, VEGF, and TNF-α decreased in human HT-29 cells stimulated with LPS and then treated with metformin. Given that these cytokines are induced in IBD, a reduction in IL-1β, IL-8, VEGF, and TNF-α mRNA levels highlights the anti-inflammatory activity of metformin. MTT assay results showed that the anti-inflammatory effects of metformin were not due to cytotoxicity.

The results from the current study demonstrate the anti-inflammatory activity of metformin in intestinal inflammation. The anti-inflammatory functions of metformin and its ability to inhibit NF-κB activation have been previously reported [36]. Our findings show that treatment with metformin prevented IBD and sustained the weight of mice. We also showed that metformin reduced the expression of several proinflammatory cytokines involved in IBD by inhibiting STAT3 phosphorylation and promoting STAT5 phosphorylation.

## Conclusion

There are few reports regarding the regulation of p-STAT3 by metformin. Our results suggest that metformin can reduce intestinal inflammation *via* the inhibition STAT3 phosphorylation and the enhancement of STAT5 phosphorylation. The activity of metformin in this study suggests that it most likely plays a key role in the suppression of intestinal inflammation. The anti-inflammatory functions of metformin could help in our understanding of the pathogenesis of immune-mediated inflammatory disorders such as IBD. Our evidence indicates that metformin might be a strong candidate for the treatment of IBD.
